# Supplementary material for: Exploring the Genetic and Functional Diversity of Porphyromonas gingivalis Survival Factor RagAB
Source: Int J Mol Sci. 2025 Jan 26;26(3):1073. doi: 10.3390/ijms26031073 (PMC11817032; doi:10.3390/ijms26031073)
Supplement: Supplementary file 1 [file ijms-26-01073-s001.zip › Supplementary Figures and Tables final.pdf]

Supplementary Figures and Tables:

Table S1: Rag-typing results of *Porphyromonas* (*P. gingivalis*, *P. gulae*) isolates subjected in this study together with strain information such as synonymous strain numbers, place and date of isolation if known and host if not human. It is notable that isolates from Germany (cities of Aachen, Freiburg, Kiel, Mainz, and Nürnberg) are disproportionately represented in our strain collection, which could introduce a potential bias.

	Type-1		Type-2		Type-3		Type-4	
1	1049	1644, AJW5 (VAG 5), USA	1047	3141, 8Pg1, Indonesia (1994)	1045	94, 376, USA	1046	3150, 21Pg1, Indonesia (1994)
2	1051	1631, 22KN6-12, Japan (Tokushima)	1056	4919, 201atPg4, Indonesia (2002)	1052	3616, CLN16-6-4, USA (Arizona)	1048	92, 382, USA
3	1053	3615, RB22D-1, Canada (Quebec)	1059	3651, THUR28BM-2, USA (Buffalo)	1057	4668, 59Pg2, Indonesia (2002)	1054	3362, 168Pg1, Indonesia (1994)
4	1060-4	3654a, JBB-c1, Sweden (Umea)	1061-1	3654b, JBB-c2, Sweden (Umea)	1058	3617, AZR6-13, USA (Buffalo)	1055	3393, 178Pg1, Indonesia (1994)
5	1068	3458, 213Pg1, Indonesia (1994)	1064	Germany (Aachen)	1076	3605, Chat2, Canada (Quebec), *P. gulae*, cat	1060-GR	3652, OMG 1426, Africa (Kenya)
6	1071*	1660, ATCC 49417, Canada (Quebec)	1066	3435, 201Pg1, Indonesia (1994)	1088*	3174, 59Pg1, Indonesia (1994)	1062-2	4941, 213Pg1, Indonesia (2002)
7	1072	3610, I-433, *P. gulae*, monkey	1067	3449, 210Pg1, Indonesia (1994)	1092	1025, A7A1-28, Netherland	1063	3646, CLN17-6-1, USA (Arizona)
8	1074	3607, 7B5, Canada (Quebec)	1069-P	4608, 8Pg1, Indonesia (2002)	1093	1349, Netherland	1077	3606, 23A4, Canada (Quebec)
9	1078-E	3200, 84Pg1, Indonesia (1994)	1070	1690, Netherland	1094	1954, Sweden	1080	3611, G251, *P. gulae*, monkey
10	1079-WHG	3196, 83Pg1, Indonesia (1994)	1073	3609, JKG5, USA (Michigan)	1097-2	2473a, T19-IIIa, Belgium (Leuven)	1102	4677, 77pg1, Indonesia (2002)
11	1081	3612, I-372, *P.gulae*, monkey	1082	184, X-2, Netherland	1098	2474, R33, Belgium	1104	4627, 21Pg1, Indonesia (2002)
12	1084	3198, 81Pg1, Indonesia (1994)	1083	319, 10-3-2007, Netherland	1124	3629, EM-3, USA (Buffalo)	1105-orange	4856, 168Pg1, Indonesia (2002)
13	1087	3261, 122Pg1, Indonesia (1994)	1085	3182, 80Pg1, Indonesia (1994)	1128	3614, Chien5B, Canada (Quebec), *P. gulae*, dog	1109	3619, BG 4, Japan (Osaka)
14	1101	4689, 83Pg1, Indonesia (2002)	1086	3320, 153Pg1, Indonesia (1994)	1152	St-Nr. 6, Germany (Kiel)	1111	3657, W, USA (Michgan)
15	1108	4685, 81Pg1, Indonesia (2002)	1089	3158, 27Pg1, Indonesia (1994)	1154	St-Nr. 7, Germany (Kiel)	1132*	ATCC 33277
16	1112-2B	3687, A 7436, USA (Georgia)	1090*	3604, HW24D-2, Canada (Quebec)	1157	St-Nr. 16, Germany (Kiel)	1160	St-Nr. 3/34, Germany (Nürnberg), *P. gulae*
17	1117	3644, W12, USA (Alabama)	1091-1	3204, 91Pg1, Indonesia (1994)	1162	St-Nr. 5/34, Germany (Nürnberg)	617	Fr. 002/23-1, Germany (Freiburg)
18	1120	3640, 13JC, France (Rennes)	1095	1691, Netherland	1163	St-Nr. 6/34, Germany (Nürnberg)	621	Fr. 016/16-1, Germany (Freiburg)
19	1122	3622, 17_5, USA (Minneapolis)	1096	3613, 19A4, Canada (Quebec)	1169	St-Nr. 12/34, Germany (Nürnberg)	625	Fr. 037/15-1, Germany (Freiburg)
20	1125	4756, 122Pg1, Indonesia (2002)	1097-3	2473b, T19-IIIb, Belgium (Leuven)	1176	St-Nr. 2, Germany (Kiel)	628	strain 381 (ATCC 33277 progeny)
21	1127	4693, 84Pg1, Indonesia (2002)	1099	3623, Y8-1, USA (Buffalo)	620	Fr. 050/13-1, Germany (Freiburg)	775	AC PJ 01, Germany (Aachen)
22	629*	W83, Germany (Bonn)	1100	3628, 332-2, Sweden (Umea)	624	Fr. 011/17-2, Germany (Freiburg)		
23			1103	4631, 27Pg1, Indonesia (2002)				
24			1106	4813, 153Pg1, Indonesia (2002)				
25			1107	4681, 80Pg1, Indonesia (2002)				
26			1110	3647, OMG 406, Africa (Kenya)				
27			1113	3630, B220-2, Sweden (Umea)				
28			1114	3631, 295-1, Sweden (Umea)				
29			1115	3636, A9A2-17, USA (Arizona)				
30			1116	3643, FAY19M-1, USA (Buffalo)				
31			1118	3645, OMZ 481, Switzerland (Zürich)				
32			1119	3637, JKG6, USA (Michigan)				
33			1121	3621, H965, Netherlands (Amsterdam)				
34			1123	4936, 210Pg1, Indonesia (2002)				
35			1126-14	4696, 91Pg1, Indonesia (2002)				
36			1129	AC 50, Germany (Aachen)				
37			1144	AC 26, Germany (Aachen)				
38			1148	AC 03, Germany (Aachen)				
39			1151	St-Nr. 1, Germany (Kiel)				
40			1153	St-Nr. 8, Germany (Kiel)				
41			1155	St-Nr. 9, Germany (Kiel)				
42			1156	St-Nr. 12, Germany (Kiel)				
43			1164	St-Nr. 7/34, Germany (Nürnberg)				
44			1192	IZKF Pat1, Germany				
45			1193-E	IZKF Pat2, Germany				
46			1194	IZKF Pat4 (Iso4), Germany				
47			1195	IZKF Pat4 (Iso2), Germany				
48			1198-X	Germany (Aachen)				
49			618	Fr. 049/26-1, Germany (Freiburg)				
50			619	Fr. 048/11-1, Germany (Freiburg)				
51			622	Fr. 025/15-1, Germany (Freiburg)				
52			731	AC 08, Germany (Aachen)				
53			743	AC 04, Germany (Aachen)				
54			772	AC 71, Germany (Aachen)				
55			778	AC 38, Germany (Aachen)				
56			779	AC 58, Germany (Aachen)				
57			782	AC 59, Germany (Aachen)				
58			784	AC 07, Germany (Aachen)				
59			883	AC 27, Germany (Aachen)				
60			884	AC 29, Germany (Aachen)				
61			887-2	AC 26, Germany (Aachen)				
62			888	MZ 04, Germany (Mainz)				
63			889-BRUC	MZ 16, Germany (Mainz)				
64			1069	Germany (Aachen)				
total:	**22**		**64**		**22**		**21**	

Table S2: NCBI references used for the mapping of our strains.

	rag-1	***rag-2***	***rag-3***	***rag-4***
ragA **references**	W83<br>W50<br>ATCC 49417	11A	SU60<br>A7A1_28	3A1<br>ATCC 33277
ragB **references**	W83<br>ATCC 49417	11A	SU60<br>A7A1_28	3A1<br>3_3<br>ATCC 33277<br>TDC60

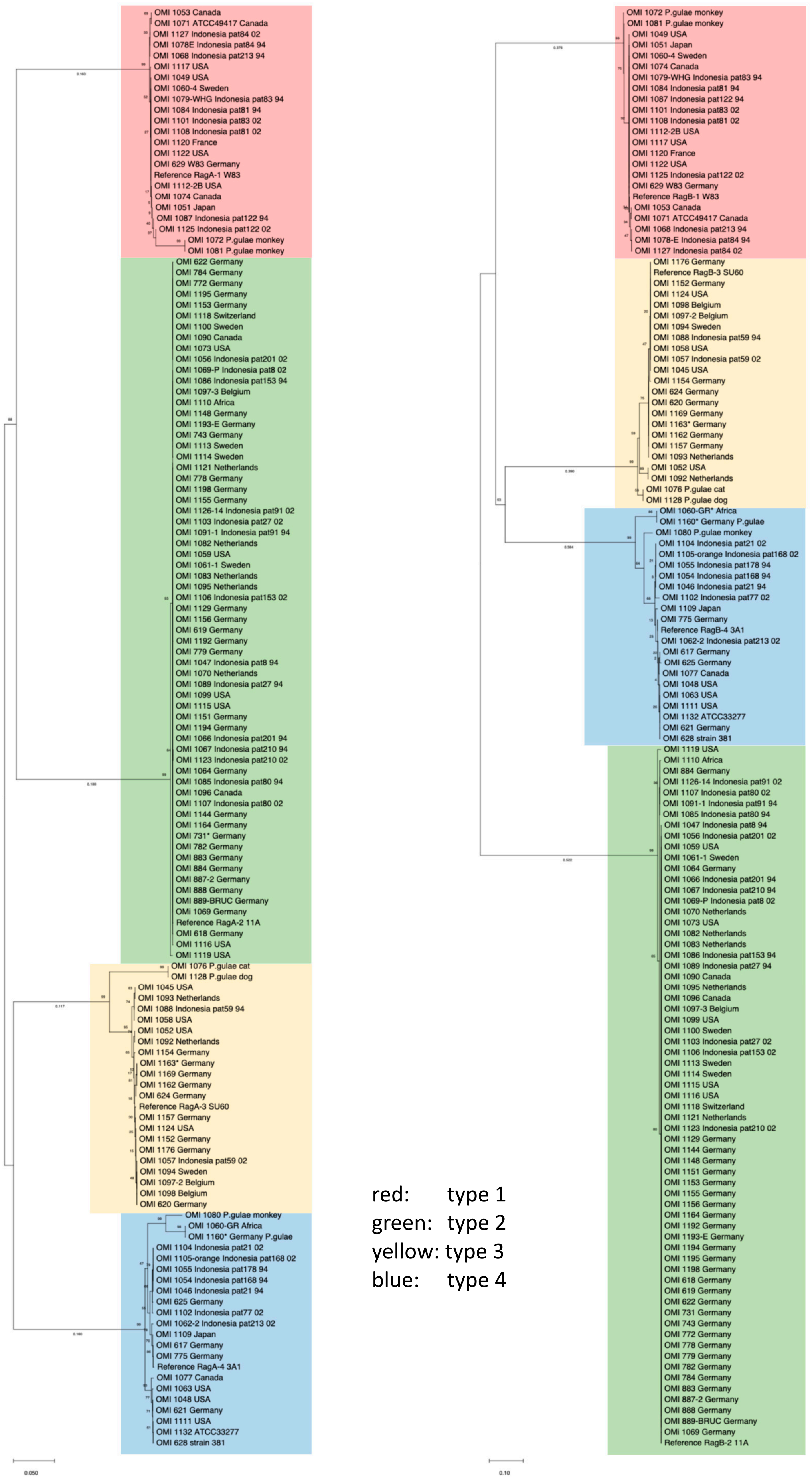

Figure S1: Neighbor-joining tree (phylograms) of *ragA* (left) and *ragB* (right).

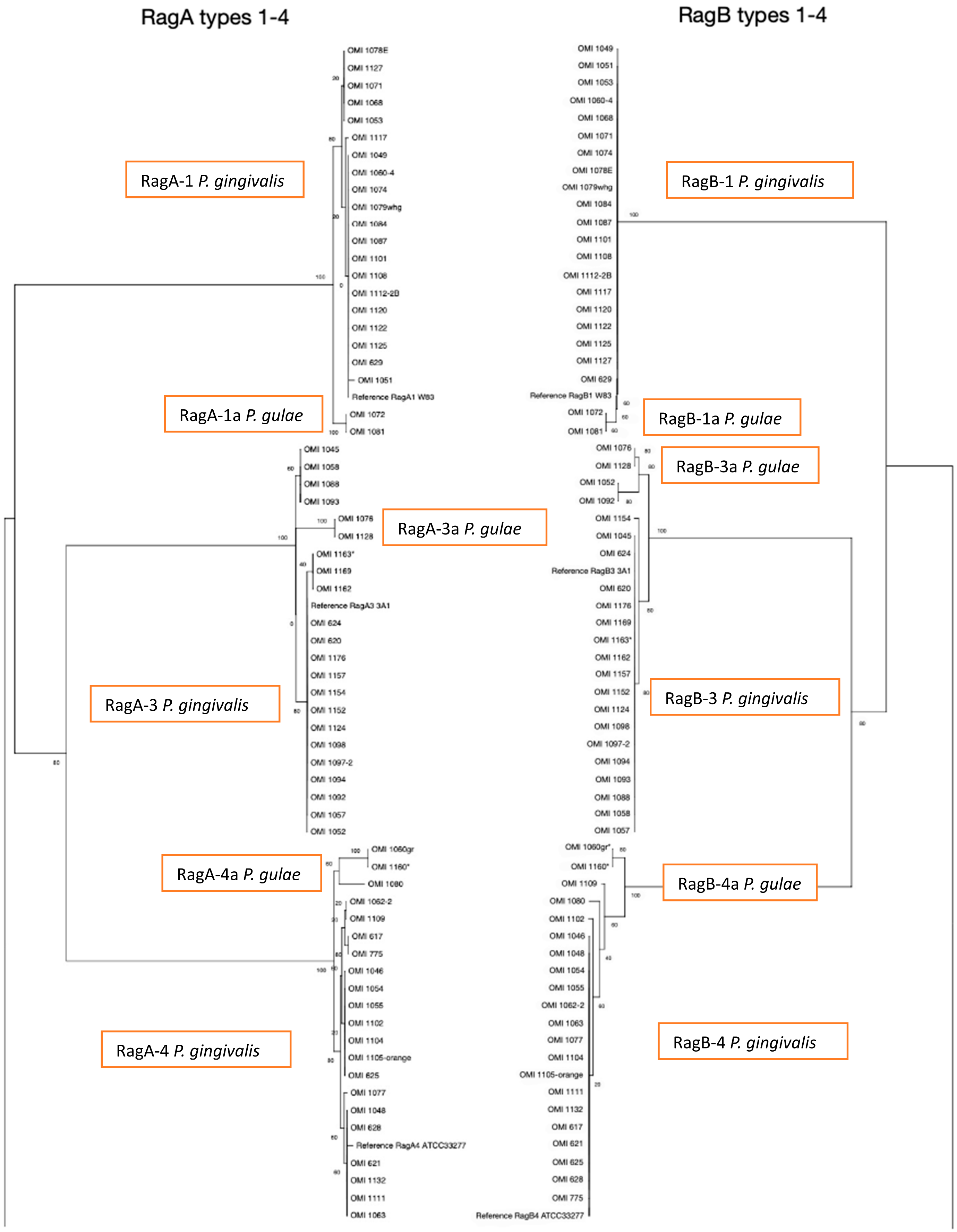
RagA types 1-4
RagB types 1-4
RagA-1 P. gingivalis
RagA-1a P. gulae
RagA-3a P. gulae
RagA-3 P. gingivalis
RagA-4a P. gulae
RagA-4 P. gingivalis
RagB-1 P. gingivalis
RagB-1a P. gulae
RagB-3a P. gulae
RagB-3 P. gingivalis
RagB-4a P. gulae
RagB-4 P. gingivalis
OMI 1078E
OMI 1127
OMI 1071
OMI 1068
OMI 1053
OMI 1117
OMI 1049
OMI 1060-4
OMI 1074
OMI 1079whg
OMI 1084
OMI 1087
OMI 1101
OMI 1108
OMI 1112-2B
OMI 1120
OMI 1122
OMI 1125
OMI 629
OMI 1051
Reference RagA1 W83
OMI 1072
OMI 1081
OMI 1045
OMI 1058
OMI 1088
OMI 1093
OMI 1076
OMI 1128
OMI 1163*
OMI 1169
OMI 1162
Reference RagA3 3A1
OMI 624
OMI 620
OMI 1176
OMI 1157
OMI 1154
OMI 1152
OMI 1124
OMI 1098
OMI 1097-2
OMI 1094
OMI 1092
OMI 1057
OMI 1052
OMI 1060gr
OMI 1160*
OMI 1080
OMI 1062-2
OMI 1109
OMI 617
OMI 775
OMI 1046
OMI 1054
OMI 1055
OMI 1102
OMI 1104
OMI 1105-orange
OMI 625
OMI 1077
OMI 1048
OMI 628
Reference RagA4 ATCC33277
OMI 621
OMI 1132
OMI 1111
OMI 1063
OMI 1049
OMI 1051
OMI 1053
OMI 1060-4
OMI 1068
OMI 1071
OMI 1074
OMI 1078E
OMI 1079whg
OMI 1084
OMI 1087
OMI 1101
OMI 1108
OMI 1112-2B
OMI 1117
OMI 1120
OMI 1122
OMI 1125
OMI 1127
OMI 629
Reference RagB1 W83
OMI 1072
OMI 1081
OMI 1076
OMI 1128
OMI 1052
OMI 1092
OMI 1154
OMI 1045
OMI 624
Reference RagB3 3A1
OMI 620
OMI 1176
OMI 1169
OMI 1163*
OMI 1162
OMI 1157
OMI 1152
OMI 1124
OMI 1098
OMI 1097-2
OMI 1094
OMI 1093
OMI 1088
OMI 1058
OMI 1057
OMI 1060gr*
OMI 1160*
OMI 1109
OMI 1080
OMI 1102
OMI 1046
OMI 1048
OMI 1054
OMI 1055
OMI 1062-2
OMI 1063
OMI 1077
OMI 1104
OMI 1105-orange
OMI 1111
OMI 1132
OMI 617
OMI 621
OMI 625
OMI 628
OMI 775
Reference RagB4 ATCC33277

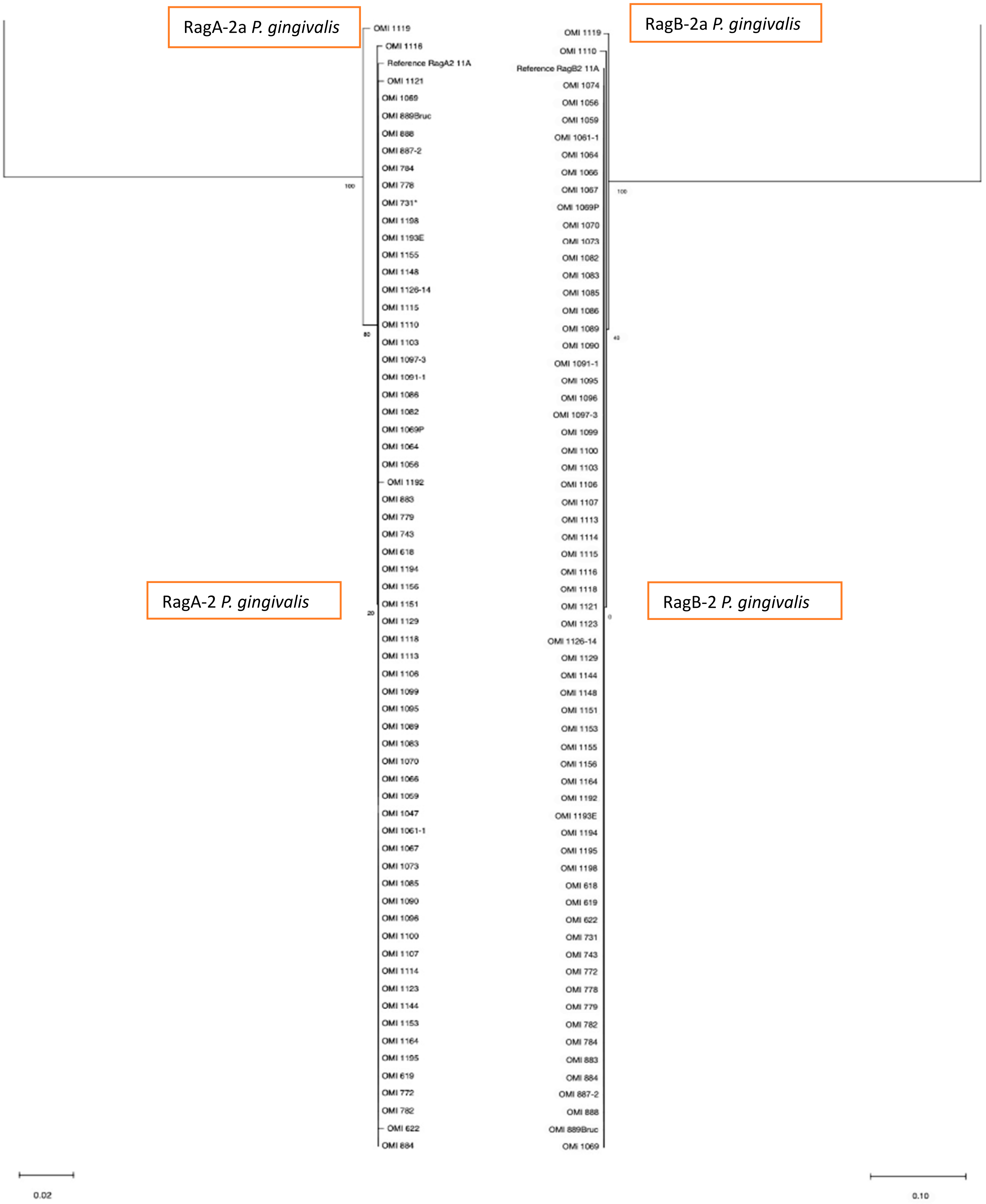

Figure S2: Neighbor-joining tree (phylograms) of RagA (left) and RagB (right) juxtaposed (100 bootstrap replications). Branches of animal isolates (*P. gulae*) become obvious.

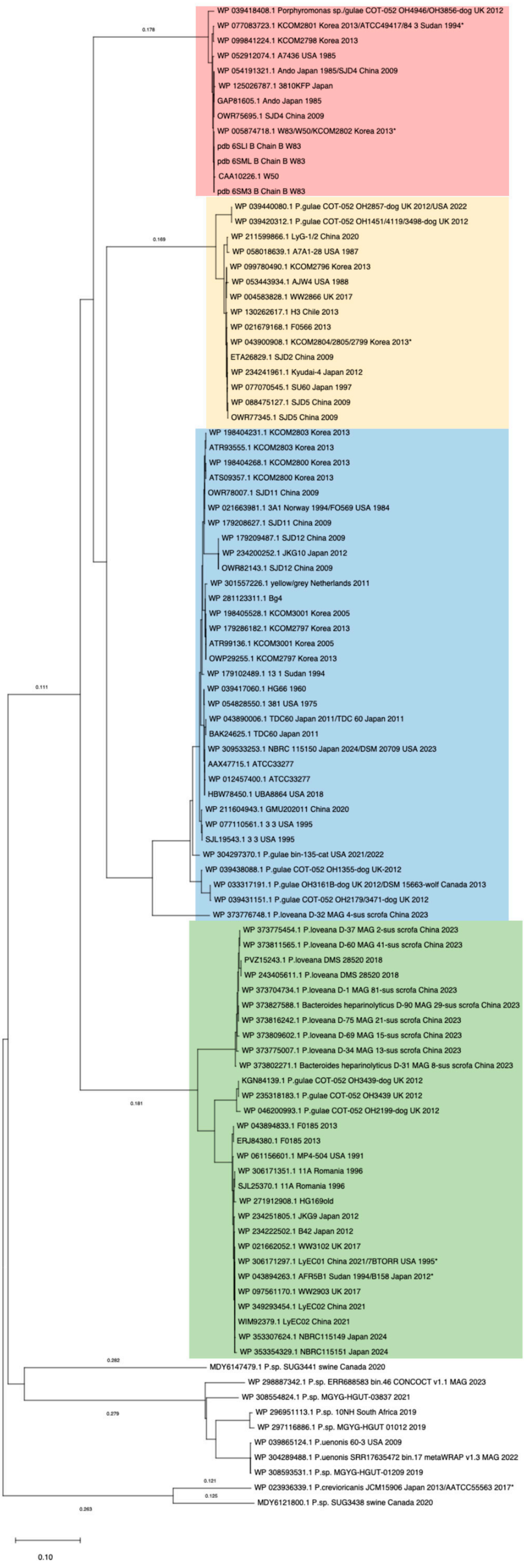

Figure S3: Phylogenetic tree (phylograms) based on 100 RagA serotypes with *Porphyromonas gingivalis* W83 as reference on top. Red: RagA-1 including *P. gulae* (1a), blue: RagA-4 including *P. gulae* (4a) and *P. loveana*, yellow: RagA-3 including *P. gulae* (3a), green: RagA-2 including *P. gulae* (2a) and *P. loveana* as well as *Bacteroides heparinolyticus*. For further contrast, more distantly related species and strains are included also (uncolored). Note that in this non-redundant database WP-numbers are representatives of a few or many different strains; for translation see **Supplementary Information** "WP-strain numbers translation".

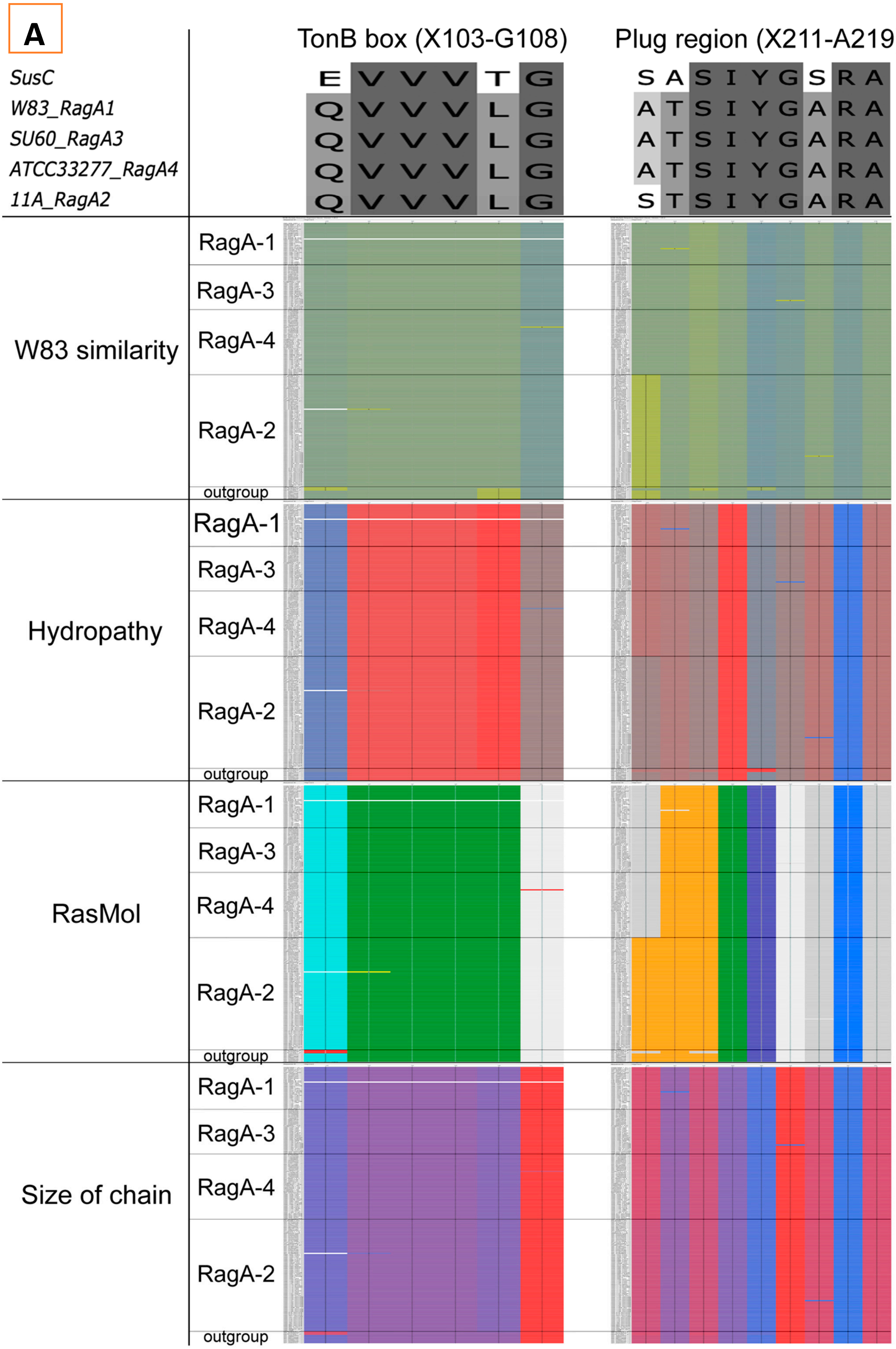
A
SusC
W83_RagA1
SU60_RagA3
ATCC33277_RagA4
11A_RagA2
TonB box (X103-G108)
Plug region (X211-A219)
E V V V T G
Q V V V L G
Q V V V L G
Q V V V L G
Q V V V L G
S A S I Y G S R A
A T S I Y G A R A
A T S I Y G A R A
A T S I Y G A R A
S T S I Y G A R A
W83 similarity
Hydropathy
RasMol
Size of chain
RagA-1
RagA-3
RagA-4
RagA-2
outgroup

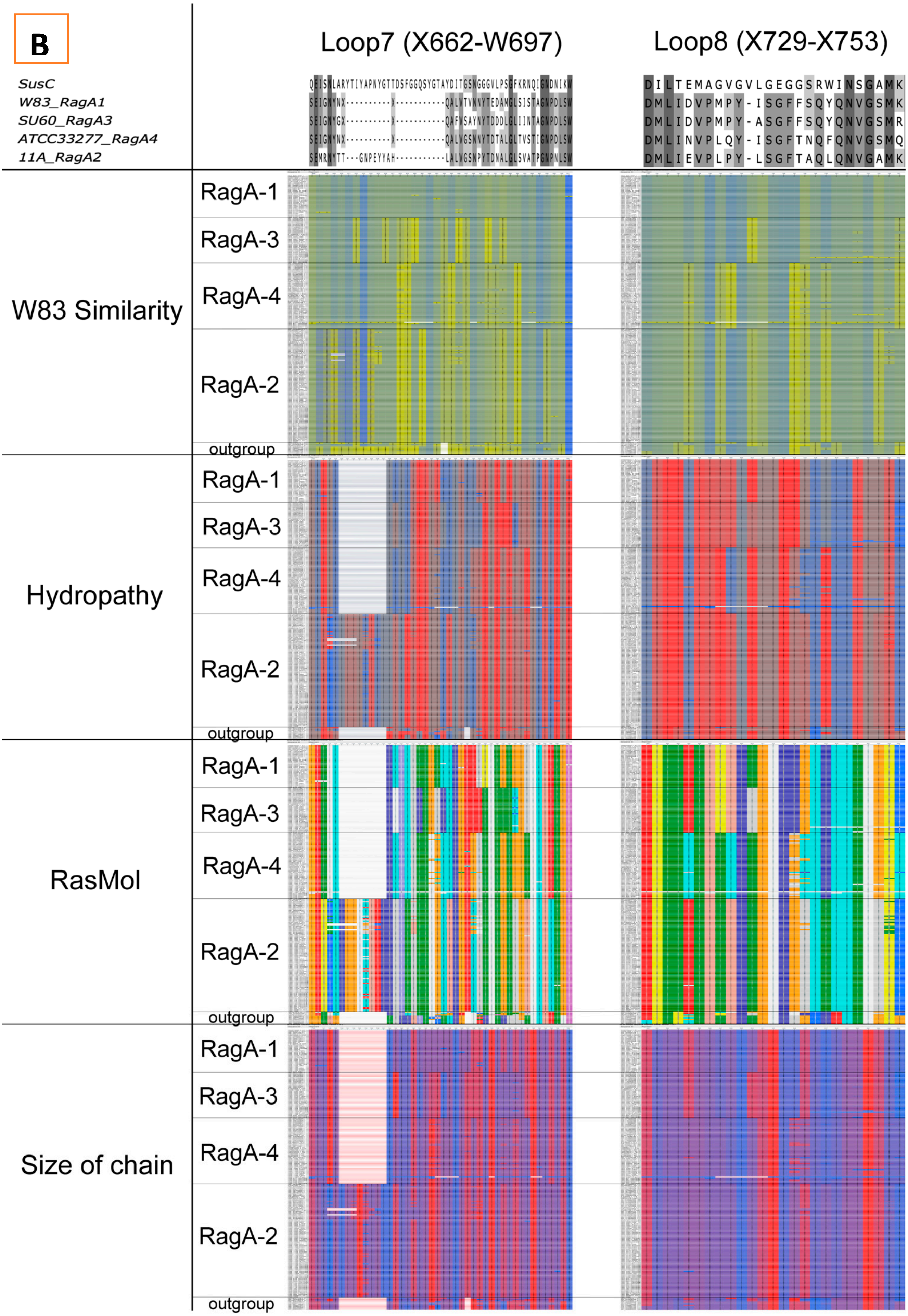
B
SusC
W83_RagA1
SU60_RagA3
ATCC33277_RagA4
11A_RagA2
Loop7 (X662-W697)
Loop8 (X729-X753)
DILTEMAGVGVLGEGGSRWINSGAMK
DMLIDVPMPY-ISGFFSQYQNVGSMK
DMLIDVPMPY-ASGFFSQYQNVGSMR
DMLINVPLQY-ISGFTNQFQNVGSMQ
DMLIEVPLPY-LSGFTAQLQNVGAMK
W83 Similarity
Hydropathy
RasMol
Size of chain
RagA-1
RagA-3
RagA-4
RagA-2
outgroup

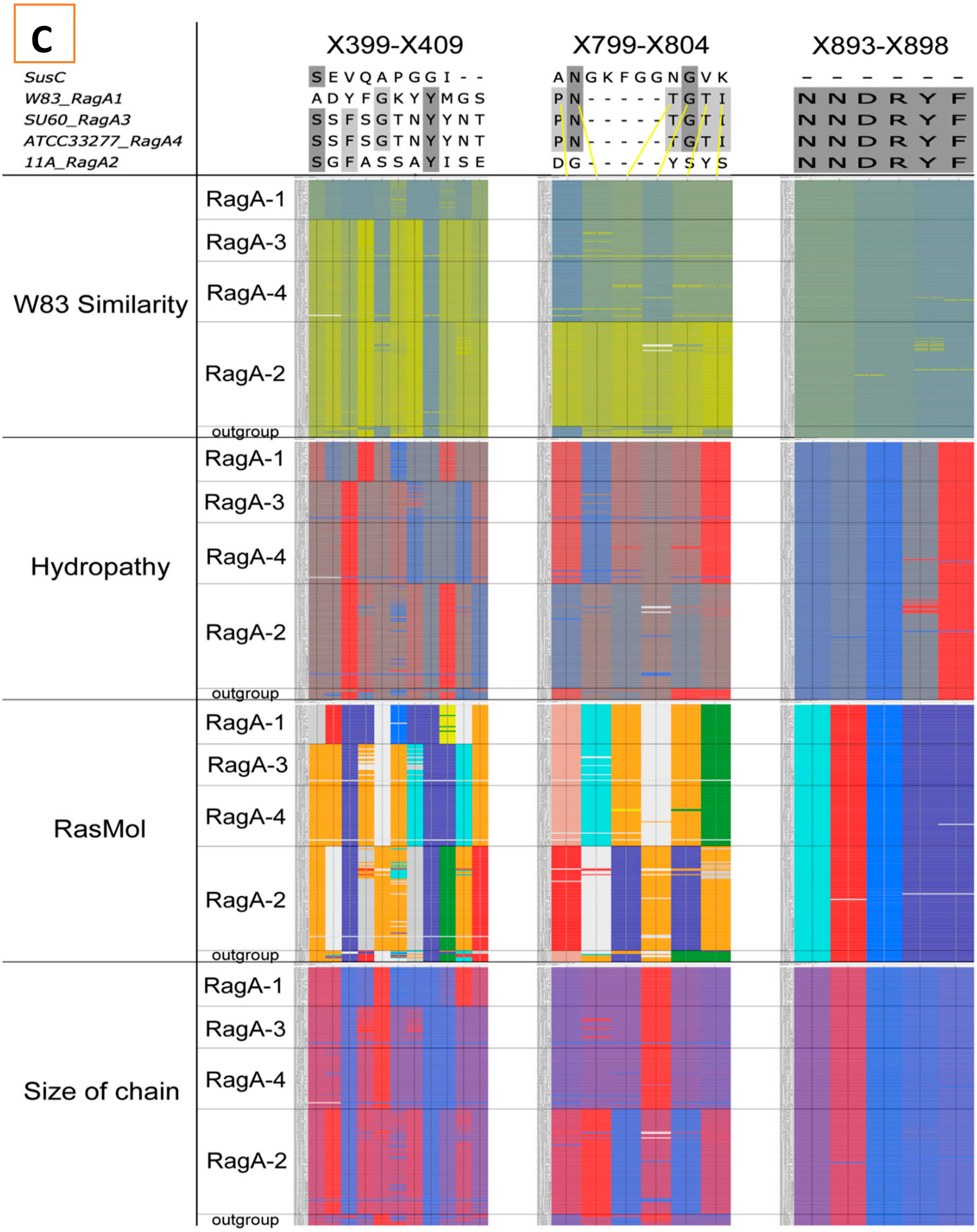

Figures S4 A-C: RagA-Mega-alignment showing differences of amino acid features (similarity to W83 as reference, hydropathy, RasMol, and size of side chain, for all of our 129 OMI- isolates and in combination with top 100 currently available RagA1-4 NCBI BLASTp results including outgroups **A:** TonB box and part of the plug region. **B:** Loops L7 and L8. **C:** potential binding regions of a proteinaceous substrate (X399-X409, X799-X804, X893-X898).

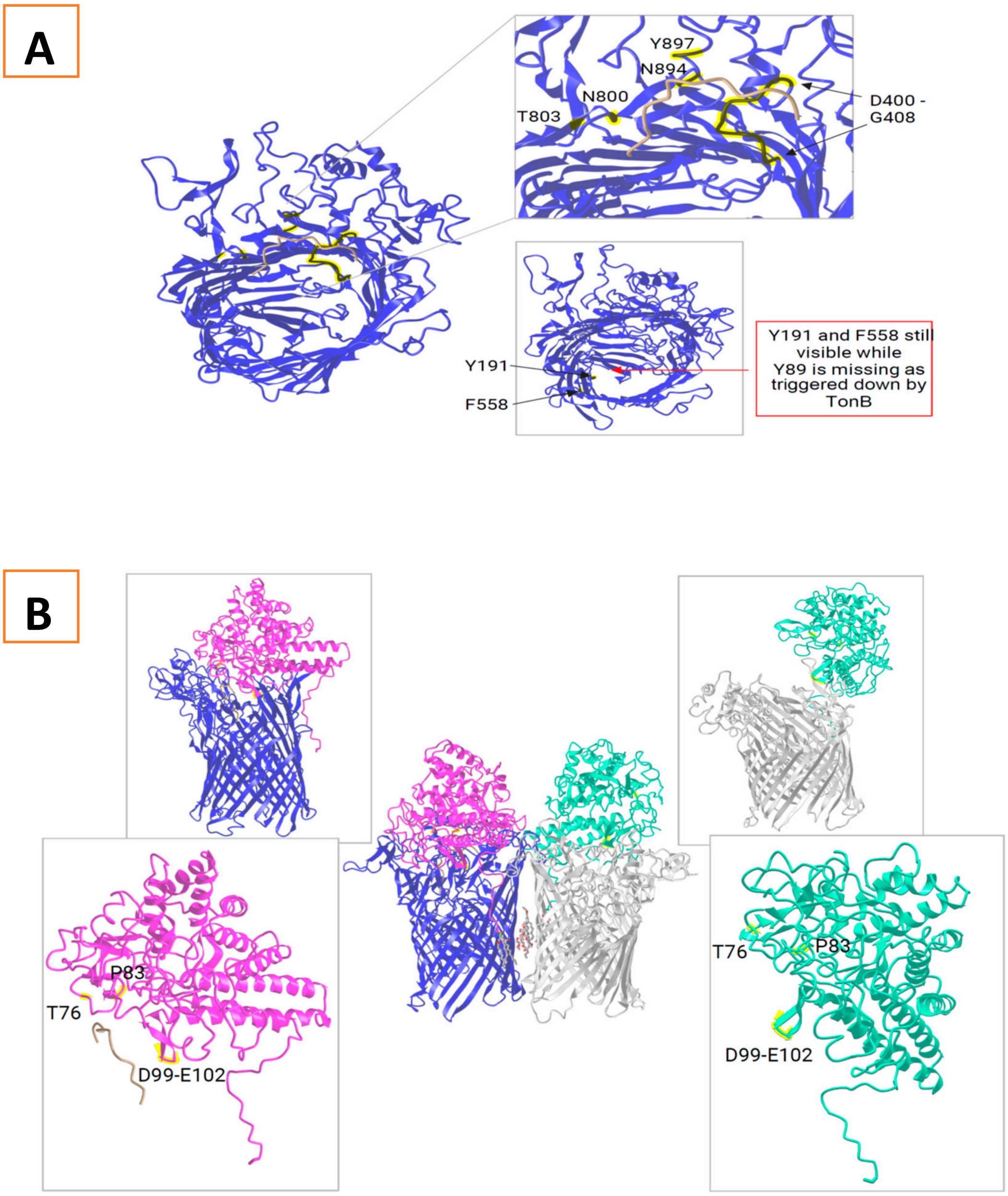

Figure S5 A-B:

A) RagA-domains in contact with the proteinaceous substrate during uptake: closed state (blue) with bound peptide (light brown); ligand-binding sites with D400-G408 as part of Loop L3, N800-T803 as part of L9 and N894-Y897 as part of L10; opposite from the aromatic stack which is now incomplete since Y89 is pulled down after TonB activation. Modified from iCn3D. Created in BioRender. Montz, P. (2025) https://BioRender.com/n62k785

B) RagB-domains in contact with the proteinaceous substrate during uptake: the open and closed state of the RagAB transporter focused on potential peptide-binding sites (T76, P83) and the acidic loop (D99-E102) of RagB type-1 (W83). On the left side the closed state (with the bound peptide) and on the right side the open (empty) state are shown. Modified from iCn3D. Created in BioRender. Montz, P. (2025) https://BioRender.com/p67e954

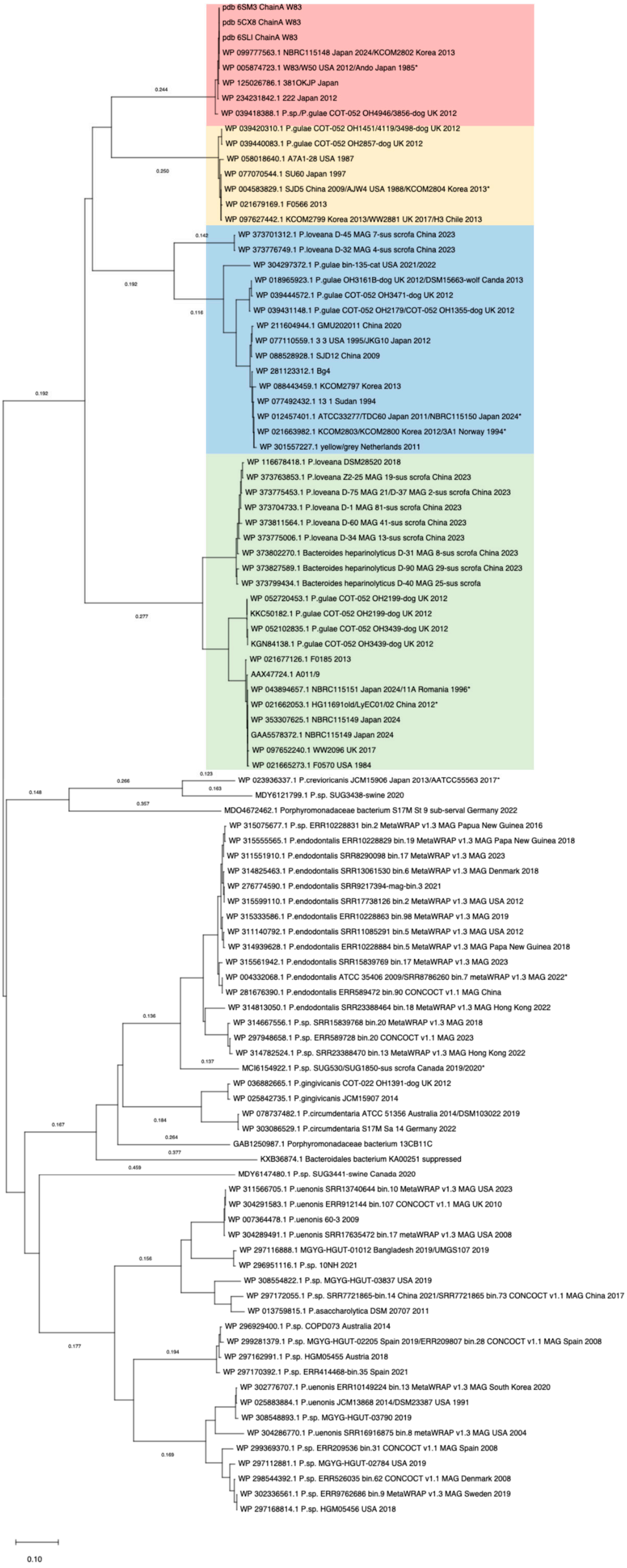

Figure S6: Phylogenetic tree (phylograms) based on 100 RagB serotypes with *Porphyromonas gingivalis* W83 as reference on top. Red: RagB-1 including *P. gulae* (1a), yellow: RagB-3 including *P. gulae* (3a), blue: RagB-4, green: RagB-2 including *P. gulae* (2a) and *P. loveana* as well as *Bacteroides heparinolyticus*. For further contrast, more distantly related species and strains are included also (uncolored). Note that in this non-redundant database WP-numbers are representatives of a few or many different strains; for translation see **Supplementary Information** "WP-strain numbers translation".

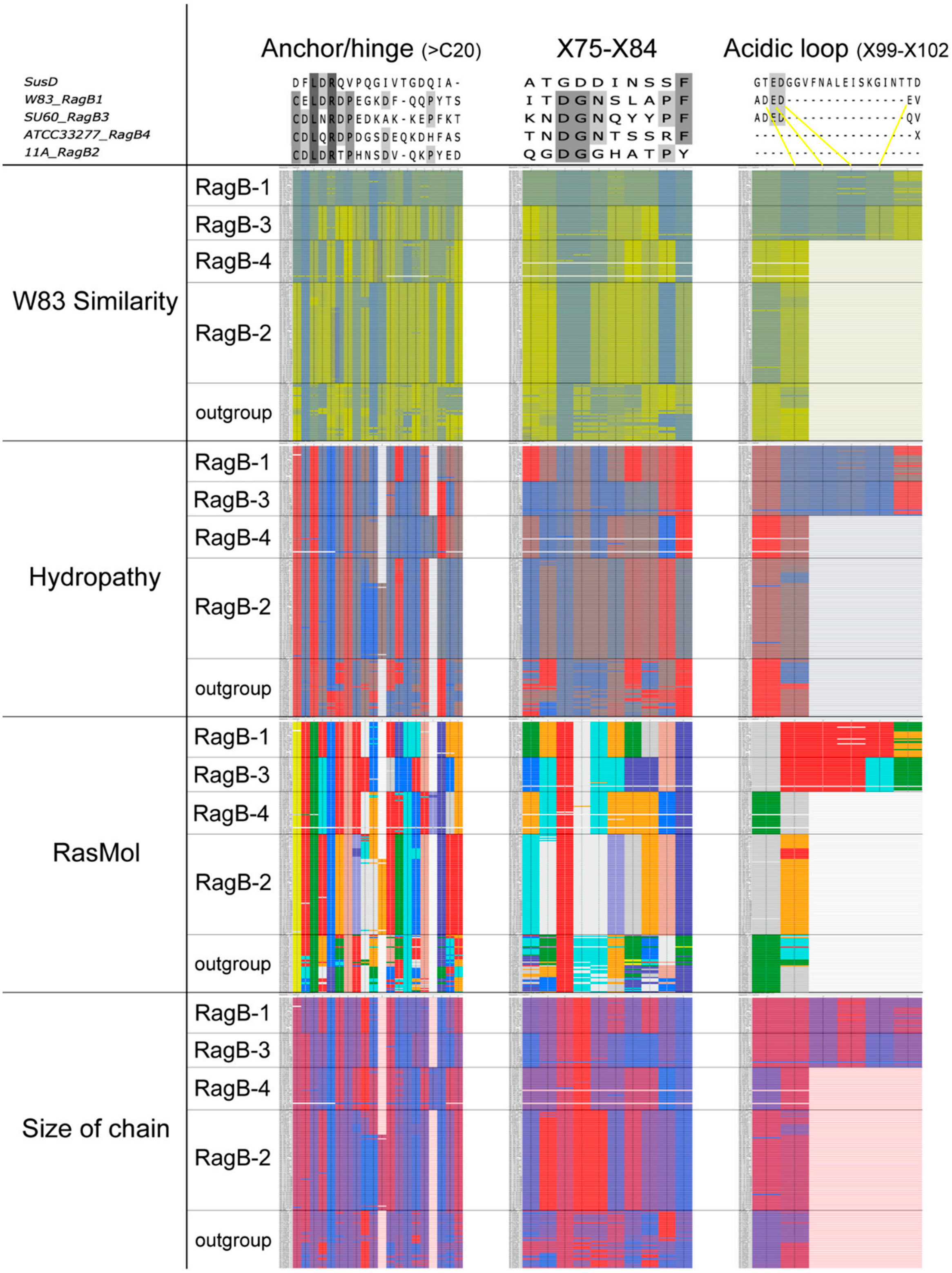

Figure S7: RagB-Mega-alignment showing differences of amino acid features (similarity to W83 as reference, hydropathy, RasMol, and size of side chain, for all of our isolates and in combination with the top 100 currently available RagB1-4 NCBI BLASTp results including outgroups. The anchor region, a potential peptide-binding region (X75-X84) and the acidic loop insertion are shown.

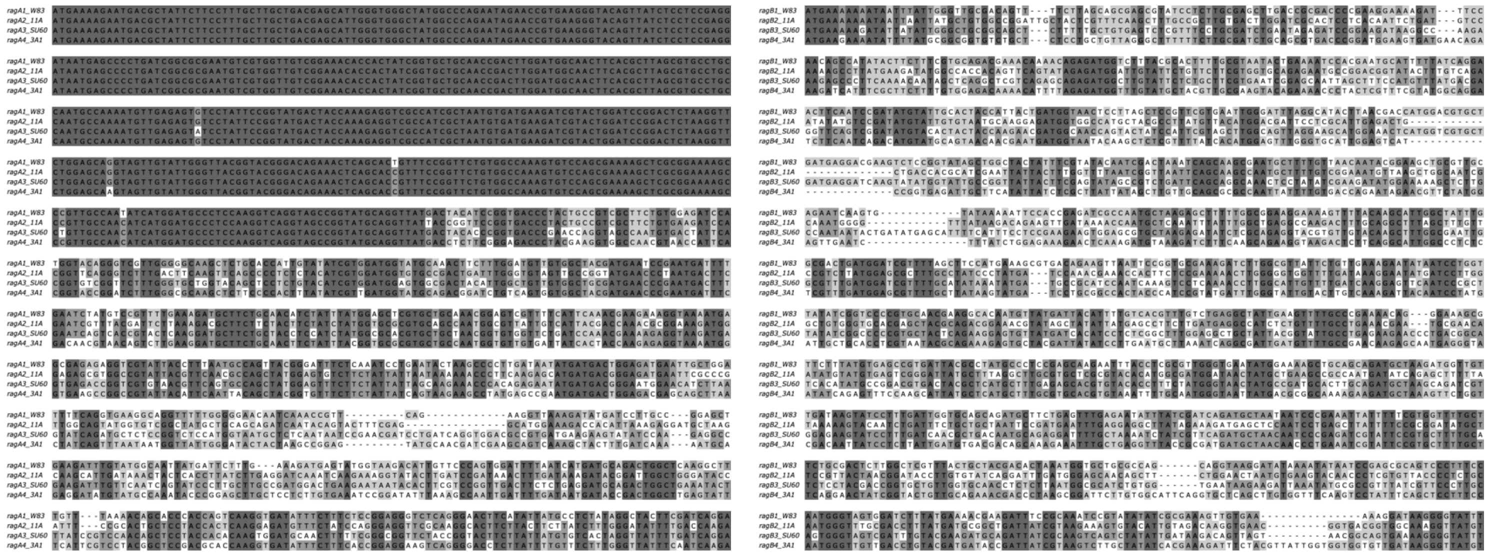

Figure S8: The partial *ragA* alignment (left) of types 1-4, ranging from bases 1-1100: the first 450 bases (N-terminus), coding a 20 aa-signal peptide and a carboxypeptidase D_ regulatory-like domain is conserved for all four types, before type-specific heterogeneity starts. Contrarily, *ragB* shows heterogeneity shortly after the N-terminal start (Unipro UGENE 50.0, similarities are shown in different grey scales, each row are at most 100b).

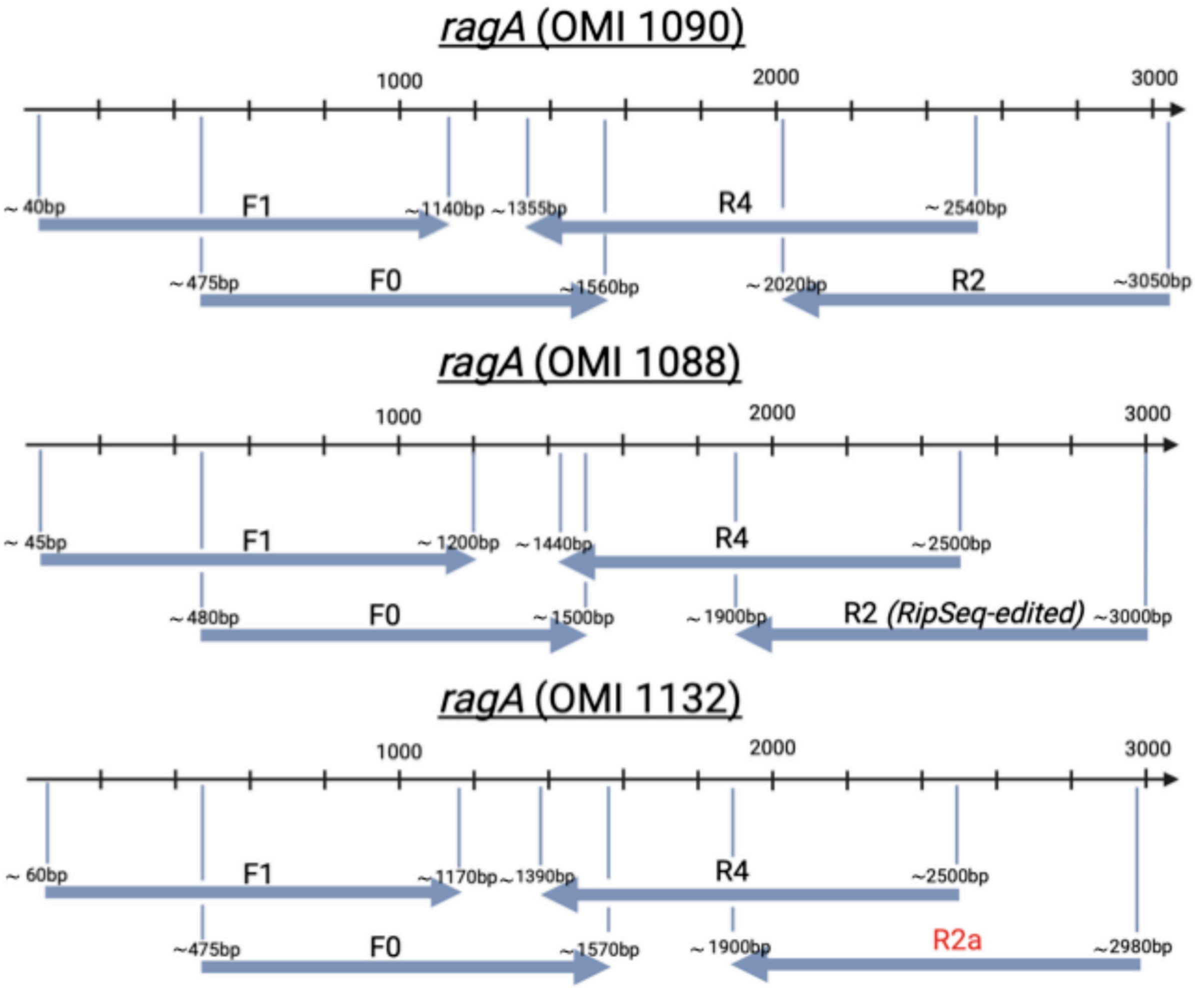

Figure S9: Sequencing strategy for *ragA-2* (OMI 1090, HW24D-2), *ragA-3* (OMI 1088, 59Pg1) and *ragA-4* (OMI 1132, ATCC 33277) complementing *ragA-1* of Figure 4. Note that this approach does not cover the first and last 50 bp of *ragA*.
